# An Investigation on the Usability of Acceleration Test by Impressed Anodic Current for Evaluating Corrosion Behavior of Hot-Dip Galvanized Rebar in Concrete

**DOI:** 10.3390/ma12213566

**Published:** 2019-10-30

**Authors:** Hong-bok Choe, Yuhei Nishio, Manabu Kanematsu

**Affiliations:** 1 Department of Architecture, Graduate School of Science and Technology, Tokyo University of Science, 2641 Yamazaki, Noda, Chiba 278-8510, Japan; 2 Department of Architecture, Faculty of Science and Technology, Tokyo University of Science, 2641 Yamazaki, Noda, Chiba 278-8510, Japan; y.nishio@rs.tus.ac.jp

**Keywords:** hot-dip galvanized rebar, impressed anodic current, applied current density, polarization resistance, zinc coating thickness

## Abstract

Hot-dip Galvanized rebar (hereafter, HDG rebar) has an anti-corrosion effect due to the sacrificial anodic reaction of zinc. Additionally, the zinc coating itself provides barrier protection for the steel substrate. Meanwhile, from one of the investigations on the field performance of HDG rebar in concrete, HDG rebar did not protect the substrate when the remaining zinc was under 50 µm. For the evaluation of this property over a short period of time, an acceleration test using impressed anodic current (hereafter, acceleration test) may be useful. This test impresses constant direct current into the rebar and can result in the intended quantitative extent of the anodic reaction. However, in using this test on HDG rebar, it was found that the high rate of applied current density could cause an unintended early end of the anti-corrosion effect of zinc, despite there being more than 50 µm of remaining zinc thickness. In this study, the usability of the acceleration test was investigated to determine if it is a suitable method for evaluating the anti-corrosion behavior of HDG rebar in concrete. As a test variable, a comparatively low rate of applied current density was used in the experiments. As a result, it was clarified that an effective corrosion protection of the substrate was made with an increase of the zinc corrosion amount. This anti-corrosion effect was similar to that known to exist in actual corrosion environments. This behavior was terminated when the concrete cracked, and the substrate became corroded. While the test condition in this study resulted in an early end of the anti-corrosion effect of zinc, a linear correlation was achieved between the applied current density and the remaining zinc thickness at the time that the anti-corrosion effect was terminated. It was found that lowering the applied current density resulted in a more suitable test condition. In conclusion, the acceleration test was found to be useful, although further experimental validation is necessary to confirm this finding.

## 1. Introduction

Currently, studies on the anti-corrosion performance of hot-dip galvanized rebar (hereafter, HDG rebar) in concrete have been carried out [1,2,3,4,5,6,7,8]. HDG rebar is a zinc-coated steel reinforcement. It is known to have an anti-corrosion effect due to the sacrificial anodic reaction of zinc. Since zinc is electrochemically a more active metal than iron, zinc corrodes itself, replacing the steel substrate and suppressing the corrosion of steel. This reaction also works on a damaged surface (exposed substrate). Furthermore, the zinc coating itself provides barrier protection for the steel substrate. For this reason, HDG rebar has been expected to have a long-term corrosion protection service life [1,2,3,4].

Concerning this anti-corrosion behavior, investigations on the field performance of HDG rebar in aged concrete have been carried out as well. On the aged concrete, which was from 3–28 years old, most of them indicated that the HDG rebar showed a sufficient amount of remaining zinc thickness, with negligible or a little corrosion of the substrate [9,10,11,12,13]. Meanwhile, from one of the investigations, which was the result at Tioga bridge in Pennsylvania (17-year-old concrete), a sample showed severe surface cracking, and the zinc thickness was 38 µm [12]. Additionally, though it was the result of a cyclic salt water wetting and drying test for 20–60 months (0.7 water/cement ratio concrete cylinder, 45–100 μm of zinc coating thickness), the HDG rebar, which was below a zinc thickness of 50 µm, showed corrosion of the substrate [14]. From this result, it was shown that the HDG rebar did not protect the substrate, when the remaining zinc was below 50 µm.

For the evaluation of this property over a short period of time, an acceleration test using impressed anodic current (hereafter, acceleration test) may be useful. This test uses a direct current (hereafter, DC) power supply to impress constant direct current into the rebar and results in an intended anodic reaction in a quantitative way. Besides, it can adjust the total current amount (mA × h/cm^2^) and applied current density (mA/cm^2^). Because of this characteristic, the acceleration test has been used to evaluate the corrosion behavior or the structural performance of ordinary rebar [15,16,17,18,19,20] and HDG rebar as well, along with the embedded state in concrete [21,22,23,24,25,26].

However, in using this test on HDG rebar, Niwa et al. [6] found that the high rate of the applied current density could cause an unintended early end of the anti-corrosion effect of zinc. In their study, 0.625 mA/cm^2^ of the current density was impressed to corrode the HDG rebar, of which the coating thickness was 153 µm. As a result, after 43% of the actual corrosion amount occurred, the substrate started to corrode, and the anti-corrosion effect did not function. At this stage, the remaining zinc thickness was 87 µm. From this result, it was found that the anti-corrosion performance of HDG rebar could be underestimated by the influence of the applied current density.

In this study, the usability of the acceleration test was investigated, considering if it is a suitable method for evaluating the anti-corrosion behavior of HDG rebar in concrete. As a test variable, the comparatively low rate of applied current density was used in the experiments. Additionally, the water/cement ratio of concrete (hereafter, w/c) and the total current amount were considered as variables. After the acceleration test, the polarization resistance and corroded amount of zinc were evaluated for comparison with previous studies.

## 2. Materials and Methods

### 2.1. Experimental Scope

Table 1 shows the specimen list and experimental variables. In this study, the w/c, applied current density, and designed corrosion amount (hereafter, DCA) were considered as variables. 

Concerning w/c, concretes were prepared with w/c = 50% and w/c = 83.5%, which allows for the investigation of whether the concrete mix proportion influences the corrosion extent of HDG rebar. When w/c = 83.5%, concrete is the same condition as that was used in the study of Niwa et al. [6].

Concerning the applied current density, 0.16, 0.08 and 0.02 mA/cm^2^ were impressed on w/c = 50% concrete. On w/c = 83.5% concrete, 0.08 and 0.23 mA/cm^2^ were impressed. 0.08 mA/cm^2^ was considered the same acceleration condition as that between w/c = 50% and 83.5% concrete. In this experiment, the current impressed by a DC power supply was 10 mA on all specimens to provide the lowest anodic current possible. Therefore, the applied current density (mA/cm^2^) was adjusted by the surface area (cm^2^) of the HDG rebar of each specimen.

Concerning DCA, 10%–100% of DCA was considered. On each specimen, 25%, 50%, 75%, and 100% were the most important test conditions. DCA indicates an estimated corrosion thickness of zinc coating, which is estimated by the total current amount (mA × h/cm^2^) of the acceleration test. A DCA of 100% indicates a full consumption of the zinc coating formed on HDG rebar. The measurement result using the thickness meter showed that 180 μm of the zinc coating was formed when it was in a non-corroded state. Therefore, 100% of DCA indicated a zinc corrosion of 180 μm, which was considered as the reference thickness. In this study, the DCA focused on the anodic reaction of zinc only, which is the stage before the corrosion of the substrate.

Equations (1) and (2) and Table 2 indicate the process of the estimation of the required total current amount for DCA in this experiment. Equation (1) is Faraday’s law [27], which explained the relationship between the corrosion current density and corrosion rate of zinc. Equation (2) was derived based on this relationship, and the (year) was converted into the (hour). Therefore, if the thickness of the zinc coating is known, the acceleration test can show the corrosion extent of HDG rebar quantitatively, as shown in Table 2. From the total current amount (mA × h/cm^2^), the acceleration test period (h) was adjusted by the applied current density of each specimen.

1 μA/cm^2^ ≈ 14.98 μm/year (1)

(Left: corrosion current density; Right: corrosion rate of zinc, calculated by Faraday’s law).

1 mA × h/cm^2^ ≈ 1.71 μm (2)

(Left: total current amount from the acceleration test; Right: corrosion thickness of the zinc coating).

### 2.2. Specimen Preparation and the Acceleration Test

**Figure 1:**
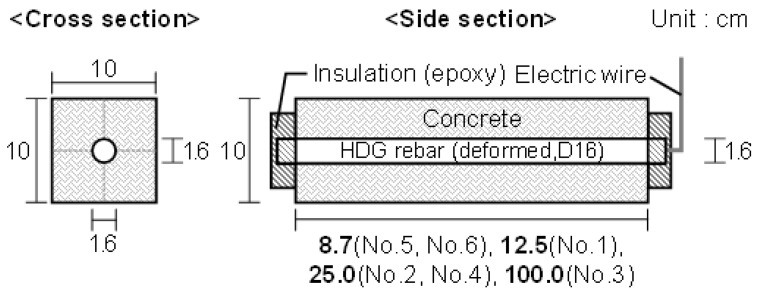
Specimen outline.

Figure 1 shows the specimen setup. Single deformed rebar was used in this experiment, and the nominal diameter was 15.9 mm (D16). On the concrete, 100 × 100 mm^2^ of the cross-section was made, and the length was prepared, considering the surface area of HDG rebar. On the cross-section of HDG rebar, an electric wire was connected to make the HDG rebar the anode in the acceleration test. After that, both sides were insulated by epoxy adhesive. Table 3 indicates the mix proportion of the concrete used for the specimen.

**Figure 2:**
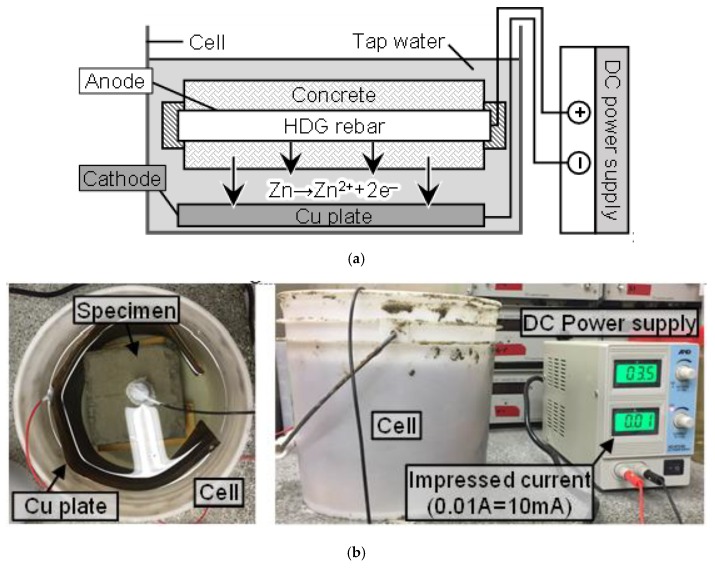
(**a**) Acceleration test; (**b**) View of the acceleration test as the current was impressed on the specimen.

Figure 2 shows the composition of the acceleration test. Firstly, a Cu plate was used as a cathode and placed on the bottom of the cell. Secondly, the specimen was immersed in tap water at a certain distance from the plate. Thirdly, the specimen was connected to the (+) terminal, and the Cu plate was connected to the (–) terminal of the DC power supply. Finally, by turning on the device to impress the constant current (10 mA), the test was initiated. While the test was in progress, tap water was periodically supplied to the cell to maintain the wet condition of the specimen.

**Figure 3:**
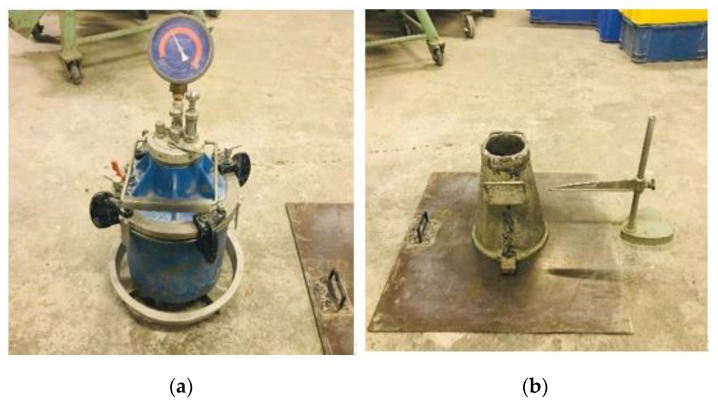
Devices used in the measurement of fresh concrete for the specimens. (**a**) Concrete air meter; (**b**) Slump test.

In Table 3, the air content (%) was measured by a concrete air meter, and the slump height (cm) and slump flow (cm) was measured by a slump cone. Figure 3 indicates the devices used in the measurement of fresh concrete for the specimens.

### 2.3. Polarization Test

**Figure 4:**
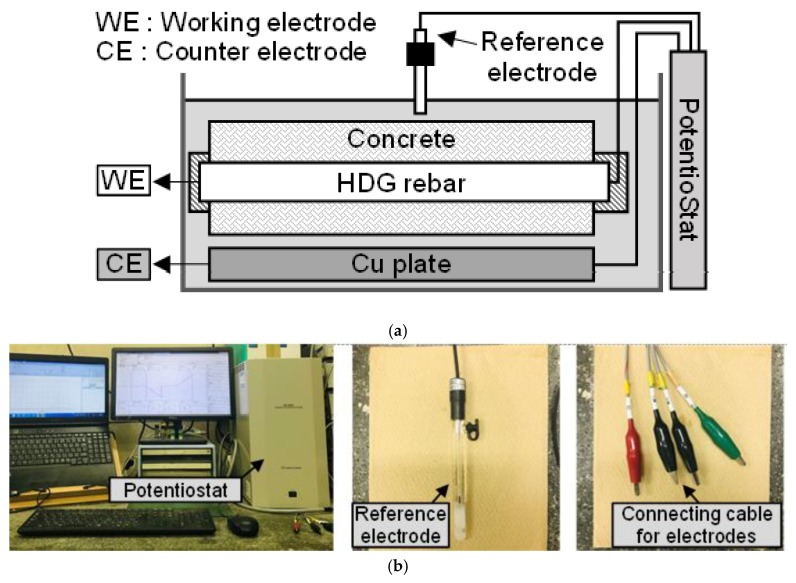
(**a**) Polarization test; (**b**) View of the polarization test outline and the main measurement devices.

Figure 4 shows an outline of the polarization test, which was carried out after the acceleration test to evaluate the electrochemical behavior of HDG rebar. Employing the potentiostatic technique, three electrodes were used, which were the Ag/AgCl electrode (reference), HDG rebar in the specimen (working), and Cu plate (counter).

Before polarization, the corrosion potential (hereafter, *E_corr_*) was measured for 30 min. *E_corr_* is defined as a potential, which is stable in its natural state. As a polarization condition, the scanning rate of the potential was 1 mV/s, the anodic polarization range was from *E_corr_* to *E_corr_* + 250 mV, and the cathodic polarization range was from *E_corr_* to *E_corr_*—250 mV. The test was carried out in the order of anodic polarization → hold of polarization (300 seconds) → cathodic polarization. When the polarization was finished, a plot of the scanned potential (hereafter, *E*) and the measured current (hereafter, *I*) (= *E − I* plot) was drawn.

After the polarization, the electrochemical properties were evaluated based on the stern-geary equation [27], as shown in Equation (3). In calculating *B*, an [*E* − *log I*] plot was used to measure the anodic gradient (hereafter, *βa*) and cathodic gradient (hereafter, *βc)*. *βa* and *βc* are located in the tafel region and generally appear as linear slopes. *βa* was measured, where the potential is over +100 mV from *E_corr_*. *βc* was measured, where the potential is over −100 mV from *E_corr_*. In calculating the polarization resistance (hereafter, *R_p_*), it is expressed as an ΔE/ΔI in [*E − I*] plot. In this experiment, *R_p_* was measured in a range from (*E_corr_* − 10 mV) to (*E_corr_* + 10 mV), which is known to show the linear slope [28,29,30,31].

*i_corr_* = *B* × *R_p_*^−1^ = {(*β_a_* × *β_c_*)/2.3 × (*β_a_* + *β_c_*)} × *R_p_*^−1^ (3)

*i_corr_*: Corrosion current density (μA/cm^2^), *B*: Tafel constant (V), *R_p_*: Polarization resistance (Ω × cm^2^), *β_a_*: Anodic gradient (V/decade), *β_c_*: Cathodic gradient (V/decade).

### 2.4. Measurement of Remaining Coating Thickness

**Figure 5:**
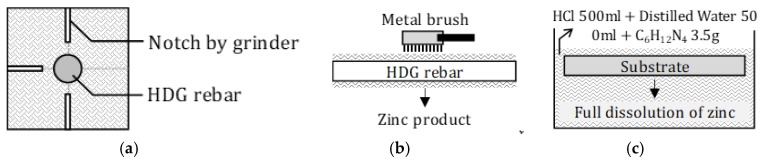
Specimen Treatment for Measuring the Remaining Coating Thickness. (**a**) Concrete cutting; (**b**) removing the zinc product and concrete on the surface (before measuring W_1_); (**c**) removal of the remaining zinc coating on the substrate (before measuring W_2_).

Figure 5 and Equation (4) indicate the process of measuring the remaining coating thickness. It compares the actual corrosion amount of zinc using DCA. Firstly, HDG rebar was taken out of the concrete. Secondly, referring to ISO 1460: 1992, weight measurements of HDG rebar, before (hereafter, W_1_) and after (hereafter, W_2_) the removal of the zinc coating, were conducted [32]. When measuring W_1_, the zinc product was removed using a metal brush. For the measurement of W_2_, a designated solution (more than 35 wt.% of HCl 500 mL + 500 mL of Distilled Water + 3.5 g of Hexamethylenetetramine (C_6_H_12_N_4_)) was used to dissolve the full amount of zinc. After that, the surface of the substrate was dried, and W_2_ was measured. Finally, the remained coating thickness (hereafter, *t_remained_*) was calculated by Equation (4).

*t_remained_* = {(W_1_ − W_2_)/7.2S} × 10^6^ (4)

*t_remained_*: Remaining thickness of zinc coating (µm), W_1_: Weight of HDG rebar, before the removal of zinc coating (g), W_2_: Weight of HDG rebar, after the removal of zinc coating (g), 7.2: Density of zinc (g/cm^3^), S: Surface area of HDG rebar (mm^2^).

## 3. Results and Discussion

Table 4 indicates the electrochemical properties and corroded amount of all specimens, after the acceleration test. *R_p_* was mainly discussed in relation to the evaluation of the corrosion behavior, and the estimated corrosion thickness (hereafter, *t_corroded_*) was also calculated using Equation (4). When calculating *t_corroded_*, the weight difference was [W_0_–W_1_]. W_0_ is the presumed weight of the uncorroded HDG rebar by its length (= 1.56 g/mm). It was measured from 15 randomly chosen samples.

### 3.1. Observation of Surface Changes of HDG Rebar

**Figure 6:**
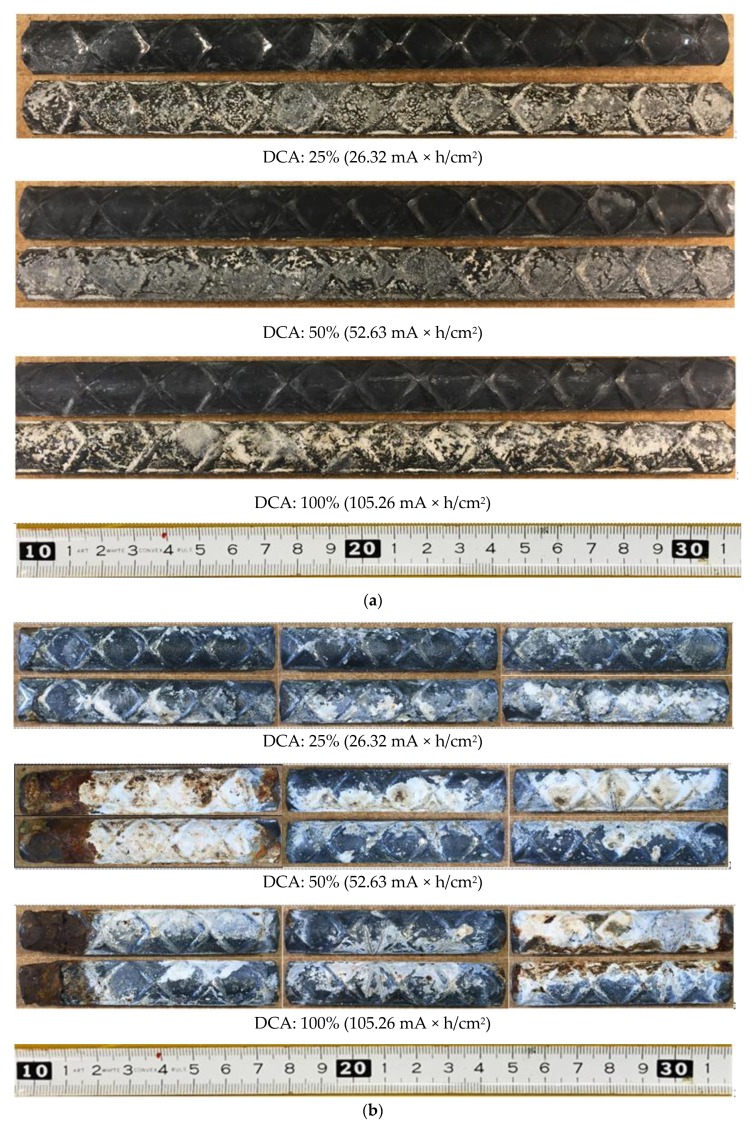
The surface of Hot-dip Galvanized (HDG) rebar, determined by an acceleration test at 0.08 mA/cm^2^. (**a**) w/c = 50%; (**b**) w/c = 83.5%.

Figure 6 shows the surface of HDG rebar in w/c = 50% and 83.5% concrete, which corroded at 0.08 mA/cm^2^ of applied current density. On each HDG rebar, the surface of both sides (top and bottom) were observed. Meanwhile, concerning other specimens that corroded at different applied current densities, the observation of the surface changes was similarly found to depend upon whether w/c is 50% or 83.5%.

From the specimens of w/c = 50% concrete, the black-colored film was formed over the entire surface of rebar, which is presumed to be an oxide film. From the observation of the cross-section, the oxide film covered the zinc coating with, for example, a thin layer. This film appeared when DCA was 25%, which is the lowest total current amount. Additionally, it was maintained in a stable condition, until the DCA was 100%. While the (silver-colored) zinc coating partially remained when DCA was 25%, this coating was unseen after DCA was 50%. Meanwhile, zinc product was mainly formed on one side of the surface by covering the oxide film, and it increased with the total current amount. However, no corrosion of the substrate was found on all specimens in w/c = 50% concrete. While the total current amount was designed to corrode the entire thickness of the zinc coating at 100% of DCA, the zinc coating remained, and thus the sacrificial anodic reaction effectively functioned.

In the specimens of w/c = 83.5% concrete, the oxide film appeared as well. However, comparatively more zinc product was formed than in w/c = 50% and was found on both sides. After that, the substrate (red product) also corroded where the zinc product was formed. Meanwhile, concerning the apparent amount of zinc product, no remarkable difference was seen between DCA = 50% and 100%. This result indicated that, when zinc product generates more than the threshold amount on a particular area, the anti-corrosion effect of zinc no longer functions, and the steel substrate starts to corrode intensively. While there was a remaining zinc coating in another area, a noticeable formation of zinc product did not occur.

Concerning the corrosion product, it is known that the representative zinc product from HDG rebar is ZnO and Zn(OH)_2_. They are loose and powdery minerals and appear as a white layer covering the surface [14,33,34,35,36]. Therefore, it was confirmed that the oxide film did not result from the reaction of zinc. Langill and Dugan [37] stated that an alloy content, such as silicon and phosphorus, is used to form a stable structure and sufficient thickness of zinc coating. In these alloy contents, silicon is a strong deoxidizing element and has a property of being combined with oxygen. Besides, in an investigation on Longbird bridge (Bermuda, 21-years-old) and Flatts bridge (Bermuda, 8-years-old), Stark et al. [9] reported that the surface of zinc coating turned black when the remaining zinc was 150 μm and 210 μm on each bridge, and both showed no corrosion of the substrate. While they were unable to determine which w/c was applied in those bridges, it is assumed that silicon in the zinc coating generated an anodic reaction to form the oxide film, irrespective of the w/c. For this reason, the corrosion of the zinc coating was delayed during the initial period of the acceleration test. Additionally, the zinc product of HDG rebar was found to be similar, when it was corroded by the acceleration test and in the actual corrosion environment. 

Concerning the corrosion behavior of HDG rebar by w/c, previous studies found that a high w/c accelerated the onset of the corrosion of HDG rebar [38,39,40,41]. Various w/c from 0.4 to 0.7 were investigated, and these studies aimed to evaluate either the corrosion current, weight loss or visual characteristics.

In the study by Clear et al. [38], slabs were cast with 0.4 and 0.5 w/c and immersed in a salt solution. As a result, the corrosion current in 0.5 w/c was 0.1 μA/cm^2^, and it was nearly twice than in 0.4 w/c (= 0.05 μA/m^2^). Additionally, Saiful Islam et al. [39] carried out an alternate wetting (using artificial seawater) and drying test with 0.4 and 0.48 w/c concrete cylinders, and the cover was 15, 25, and 40 mm. Specimens were exposed from 3 to 18 months. It was found that the weight loss of HDG rebar in 0.48 w/c was 0.12%, 0.095% and 0.032% at 15, 25, 40 mm covers. In 0.4 w/c, the weight loss was 0.11%, 0.08% and 0.038% with the same covers, which was a slightly reduced corrosion amount from 0.48 w/c. Meanwhile, Thangavel [40] investigated the corrosion resistance of HDG rebar (95 μm zinc thickness) with low-quality (0.68 w/c) and high-quality (0.5 w/c) concrete. Concrete that was chloride-contaminated and exposed by the full immersion of potable water and seawater was used for three years. The result found that 0.68 w/c concrete caused a rapid dissolution of zinc and the early corrosion of the substrate. Furthermore, Griffin [41] tested the concrete walls with 0.644 and 0.702 w/c, and the walls were approximately 1 m^2^, with a 25 mm cover. They were sprayed daily with seawater for 3 years. The result showed that, although 0.644 w/c inhibited the corrosion of HDG rebar, unlike 0.702 w/c, HDG rebar did not have a better anti-corrosion effect than non-galvanized rebar. 

Therefore, based on previous studies and the result found in this experiment, it is expected that a high ratio of w/c concrete made HDG rebar corrode more quickly and terminated the anti-corrosion effect of the zinc coating. Additionally, it is expected that the corrosion behavior in w/c = 50% is closer to that of Longbird and Flatts bridges, which were referred to in this study. While there was a difference in the corroding extent, according to the w/c, the corrosion behavior of HDG rebar was similar and occurred in the following way: the initial dissolution of a certain amount of zinc → formation of the oxide film → increase of the zinc product to the entire surface → corrosion of the substrate.

### 3.2. Electrochemical Properties

**Figure 7:**
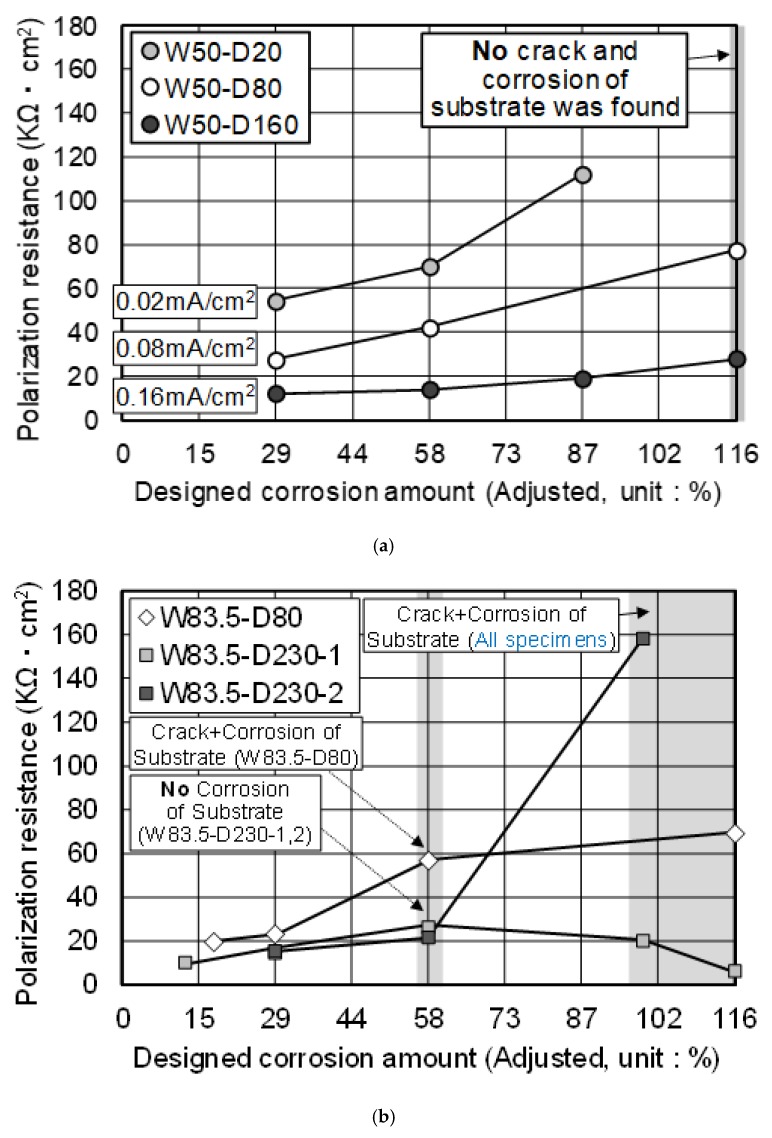
(**a**) Behavior of *Rp* (w/c = 50% concrete); (**b**) Behavior of *R_p_* (w/c = 83.5% concrete).

**Figure 8:**
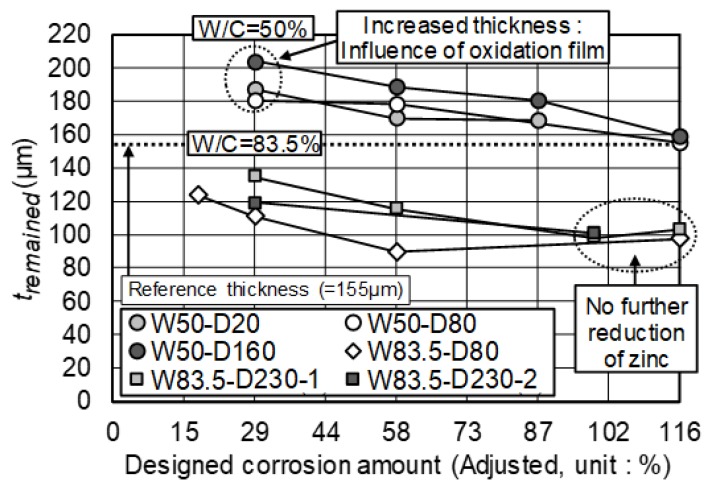
Remaining thickness of the zinc coating.

**Figure 9:**
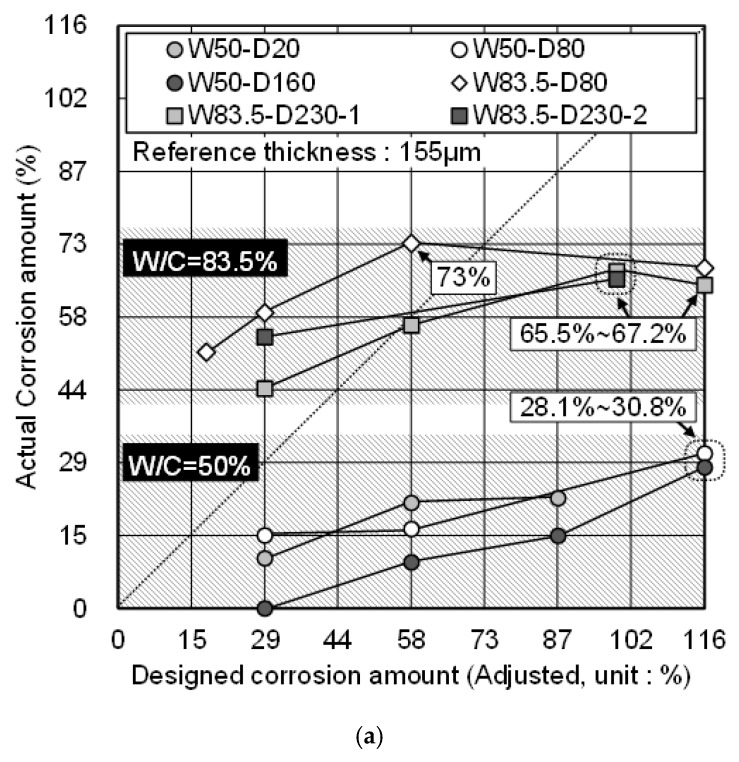

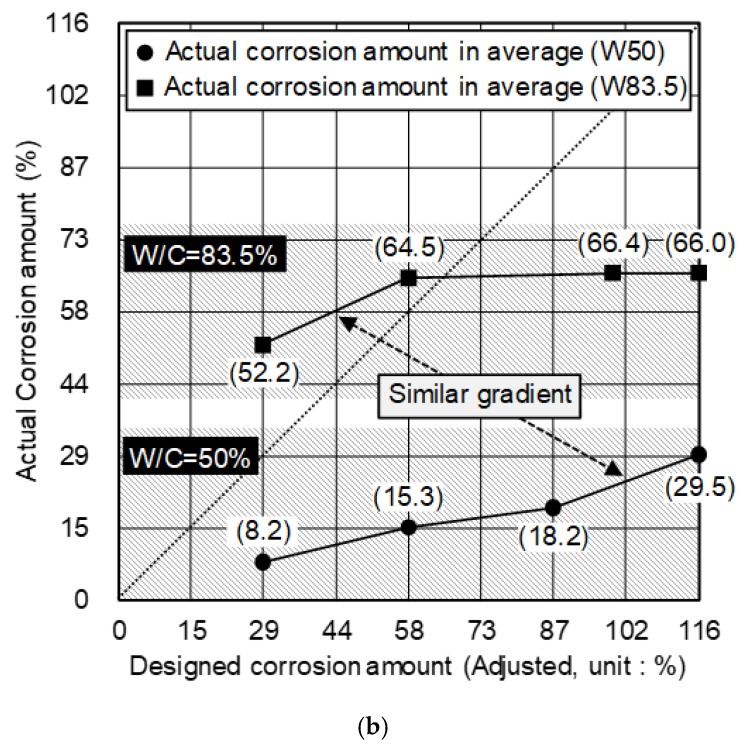
Comparison of the actual and designed corrosion amounts. (**a**) Result for each specimen; (**b**) Average.

Before discussing the experiment results, it was found that some data were scattered from the general tendency of the corrosion behavior. It is presumed that this result was due to the influence of some HDG rebar being damaged on its surface, while the fresh concrete was poured. For this reason, although specimens were prepared under the same condition, non-uniform corrosion was unexpectedly generated during the acceleration test. For this reason, Figure 7, Figure 8 and Figure 9 presented the test results, showing a constant tendency.

Figure 7 indicates the behavior of *R_p_* in all specimens. Before discussing the corrosion behavior, the adjustment of DCA was considered because of the measurement difference in the reference thickness. It was 180 µm, when measured by the thickness meter. However, when the weight difference was measured using [W_0_ − W_2_], it was calculated and found to be 155 µm by Equation (4). In this study, it was decided to adjust the reference thickness to 155 µm. This is the coating thickness, measured by the same method for evaluating the actual corrosion amount and DCA. Thus, if the reference thickness changed from 180 µm to 155 µm, DCA is increased to 1.16 times this value, because the total current amount was provided for corroding 180 µm of zinc. For example, 100% of DCA is adjusted to 116%. The adjusted DCA is presented in Figure 7, Figure 8 and Figure 9.

In the specimens of w/c = 50% concrete, *R_p_* gradually increased with the DCA. Besides, when the DCA is the same, *R_p_* was higher when the HDG rebar was corroded in the lower applied current density. In the measurement of *R_p_*, the detected current is the reaction of the surface of the metal. Thus, if a substance that shows less conductivity is formed on the surface, *R_p_* tends to be increased. In Figure 6, the zinc product became denser, as the DCA was increased. When the DCA was the same, it was observed that the W50-D20 specimen showed comparatively more zinc product on the surface.

Yeomans [33] reported that the zinc corrosion products could migrate away from the rebar and into the adjacent concrete matrix, where they fill voids and microcracks. He also stated that zinc product causes very little physical disruption to the surrounding matrix, even though the active corrosion of the coating may be occurring. Additionally, Fratesi [42] suggested that the presence of these corrosion products and the filling of the pore space in the matrix may create a barrier in the matrix with reduced permeability. By referring to the previous studies, mentioned above, it is assumed that the zinc product mainly influenced the behavior of *R_p_* in this experiment. The increasing amount of zinc product on the surface acted as a substance that resulted in a high *R_p_* in the measurement result. Additionally, concerning the difference in the applied current density, the migration of zinc product affected its formation behavior. That is to say, the lower the applied current density, the more the zinc product remained on the surface. Meanwhile, it was observed that ZnO, one of the zinc products, has no passivating properties [35,36]. However, whether the zinc product is ZnO or Zn(OH)_2_ was not determined in this study. Instead, in order to confirm if the increasing *R_p_* is related to the suppression of zinc corrosion, an evaluation of the actual corrosion amount was carried out. 

In the specimens of w/c = 83.5% concrete, a gradual increase of *R_p_* appeared, until DCA was 58%. Additionally, *R_p_* was shown to be higher at a lower applied current density. This tendency was similar to the behavior in w/c = 50% concrete. However, after DCA = 58%, the specimens showed an unstable *R_p_.* There was a decrease of *R_p_* in the W83.5-D230-1 specimen or a sharp increase of *R_p_* in the W83.5-D230-2 specimen, although these were identical samples. The W83.5-D80 specimen showed a decreased *R_p_* slope.

In this stage, a longitudinal crack on the concrete surface was found, which broke the cross-section. This crack was due to the corrosion of the substrate and expansion of the rebar. It occurred mostly after DCA was 58%, and all of the specimens showed a crack at 99% DCA. Therefore, it is assumed that the anti-corrosion effect of HDG rebar due to zinc coating was terminated at 58%–99% DCA. Additionally, while the increasing *R_p_* shows a gradual slope, the sacrificial anodic reaction of zinc is expected to be functioning effectively.

### 3.3. t_remained_ on HDG Rebar

Figure 8 indicates the *t_remained_* of all specimens, after the acceleration test. Meanwhile, in measuring *t_remained_*, W_1_ included the oxide film due to its adhesiveness. After the removal of the zinc product, it was uneasy to remove the oxide film using a metal brush. For this reason, *t_remained_* included the thickness of the oxide film as well. In this graph, the thickness of the non-corroded zinc coating was presented as the reference (= 155 µm).

From the specimens of w/c = 50% concrete, *t_remained_* was found to be higher than the reference thickness, but it decreased with the DCA. Besides, when DCA reached 116%, the *t_remained_* of all specimens were almost similar, regardless of the applied current density. On the other hand, in w/c = 83.5% concrete, *t_remained_* was lower than the reference thickness, although it included the oxide film. Additionally, when the decrease of *t_remained_* reached 100 µm at DCA = 99%, despite the increase of DCA, there were no more noticeable thickness reductions.

The result of *t_corroded_* was considered to indicate the behavior of *t_remained_*. The value of *t_corroded_* indicates that (+) is a decrease and (–) is an increase of the thickness, in relation to the initial thickness. From the result of w/c = 50% concrete, the increase of thickness at DCA = 29% was −40.33~−54.29 µm for each specimen. This increase was due to the oxide film, and it was mostly the maximum. Therefore, it is assumed that the oxide film was fully formed, before the active corrosion of zinc, as a delaying effect in the initial stage, and it is maintained at a constant thickness. In w/c = 50% concrete, the influence of the oxide film appeared when DCA = 29%. In w/c = 83.5% concrete, the delaying effect was assumed to exist when DCA was less than 29%. Therefore, referring to *t_corroded_*, which appeared in w/c = 50% concrete at DCA = 29%, it is predicted that the thickness of the oxide film is 47.64 µm, regardless of the w/c.

Meanwhile, in the scope of this study, the difference in the applied current density barely showed the influence on *t_remained_*. It was mainly affected by the total current amount. Thus, if HDG rebar was embedded in the same w/c concrete and had a certain amount of the total amount of corrosion, the actual corrosion amount was expected to be similar. Moreover, from the result of w/c = 83.5% concrete, even though *t_remained_* did not reach 0 µm, the early end of the anti-corrosion effect was confirmed. This result corresponded well with the surface change of HDG rebar, which was discussed in Figure 6. Additionally, it was clarified that the increase of *R_p_*, which showed a gradual slope in Figure 7, did not suppress the corrosion of zinc. The behavior of *R_p_* was only relevant to the property of the zinc product.

### 3.4. Comparison of the Actual Corrosion Amount and the DCA of Zinc

Figure 9 shows a comparison of the actual corrosion amount and the DCA of all specimens. The actual corrosion amount was calculated by Equation (5), which considered the predicted thickness of the oxide film and the reference thickness. [*t_remained_* − 47.64 µm] was chosen as a remained coating thickness of zinc, and 155 is the reference thickness (µm) of HDG rebar, which is not a corroded condition. In order to avoid a confusion of expression related to the corrosion amount, the (actual corrosion amount) is hereafter referred to as “ACA”.

Actual corrosion amount (%) = {1 − (*t_remained_* − 47.64)/155} × 100 (5)

In w/c = 50% concrete, the maximum actual corrosion amount (ACA) was about 29.5% against 116% of DCA. The ACA of zinc was lower than DCA over the entire period of the acceleration test. Besides, the average of all specimens at each DCA mostly showed an ACA that was about 1/4 times lower. Therefore, in the scope of this study, the HDG rebar in w/c = 50% concrete effectively protected the corrosion of the substrate and showed a constant anti-corrosion effect. 

In w/c = 83.5% concrete, the ACA of zinc was higher than the DCA during 0%~ 58%. The reason was that the corrosion reaction quickly proceeded during 0%~29%. After 58% of DCA, the corrosion of zinc did not proceed noticeably and showed the end of the anti-corrosion effect. While there was a reduction of ACA on W83.5-D80 (DCA = 58% → 116%) and W83.5-D230-1 (DCA = 99% → 116%) specimens, it was regarded as an error due to the condition, which tested only a single specimen. Therefore, it is expected that the HDG rebar in w/c = 83.5% concrete reached the threshold amount of zinc corrosion, and it was about 66%.

Meanwhile, in w/c = 50% concrete, ACA did not reach the end of the anti-corrosion effect in this study. However, during 29%–58% of DCA in w/c = 83.5% concrete, the gradient of the average ACA appeared to be similar to that in w/c = 50% concrete, when DCA = 87%–116%. This result indicates that the corrosion rate becomes constant at least after about 50% of ACA, even though w/c is different. From this result, unless the HDG rebar in w/c = 50% concrete shows a rapid increase of ACA, it is assumed that the anti-corrosion effect of zinc will continue until about 66% of ACA. By referring to the experiment result in this study, a future study is required to impress more of the current amount, until the corrosion of the substrate appears, in order to clarify the threshold amount of zinc corrosion.

### 3.5. Correspondence between the Field Performance and the Experimental Result of the Acceleration Test

In the experimental result of this study, specimens of w/c = 83.5% concrete reached the end of the anti-corrosion effect. The W83.5-D80 specimen showed 73% of ACA, and W83.5-D230 specimens showed 66% of ACA, when it was at the maximum. Meanwhile, in the study of Niwa et al. [6], ACA was 43% when corrosion of the substrate occurred. This result was tested using the acceleration test. Additionally, from the field performance of HDG rebar in Tioga bridge, the remaining zinc thickness was 38 µm when the substrate was corroded [12]. In order for the acceleration test to be useful in the evaluation of HDG rebar, it is necessary to confirm that the experiment results correspond with the result in the actual corrosion environment. Accordingly, the remaining zinc thickness at the stage when HDG rebar showed corrosion of the substrate was evaluated at each current density of the acceleration test. This is because the initial zinc thickness can differ, according to the type of HDG rebar. For this reason, how to predict the anti-corrosion performance by ACA is unclear.

**Figure 10:**
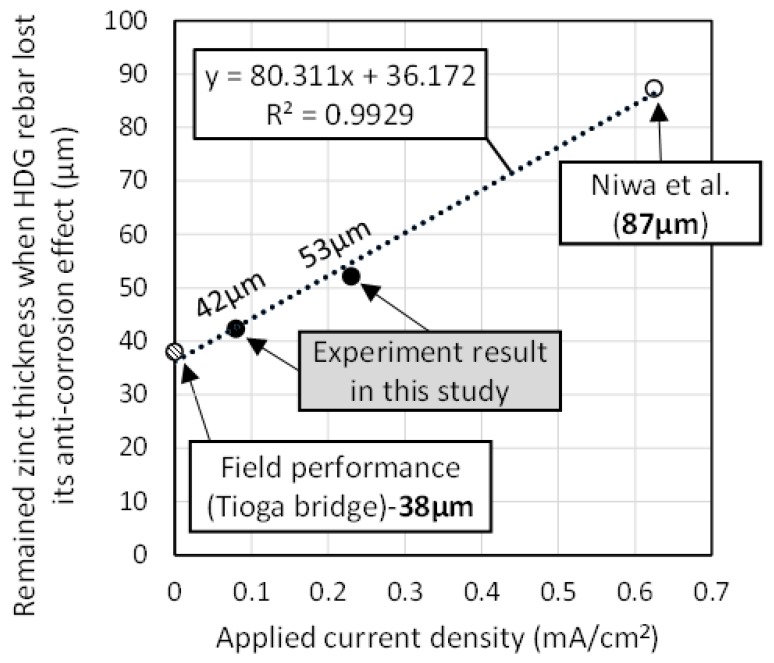
Correlation between the applied current density and the remaining zinc thickness in each corrosion environment (the acceleration test and the field performance).

Figure 10 indicates the correlation between the applied current density and the remaining zinc thickness. In this graph, the result for the field performance was regarded as not being corroded by the impression of the applied current density (=0 mA/cm^2^). Concerning the results achieved in this study, 73% of ACA at 0.08 mA/cm^2^ was converted to 42 µm of the remaining zinc thickness (= 155 µm × 0.23). In the same way, 66% of ACA at 0.23 mA/cm^2^ was converted to 53 µm (= 155 µm × 0.34). Concerning the result of Niwa et al., 43% of ACA at 0.625 mA/cm^2^ was converted to 87 µm (=153 µm × 0.57).

As a result, the regression equation showed a well-fitted correspondence between the acceleration test and the actual corrosion environment in terms of the remaining zinc thickness, which was a linear relationship. Moreover, the extent of the applied current density was influential in causing the early end of the anti-corrosion effect. It is expected that lowering the applied current density will result in a more suitable test condition. In this graph, it is recommended that the acceleration test needs to be carried out below 0.1 mA/cm^2^. From this result, the acceleration test was found to be useful. 

Meanwhile, concerning the data referred to in this study, it was not widely investigated whether this linear relationship is expected to appear in the range of 0.23 mA/cm^2^–0.625 mA/cm^2^. Therefore, in order to clarify a more suitable range for the acceleration test condition, further experimental validation at various applied current densities is necessary.

## 4. Conclusions

In this study, the usability of the acceleration test was investigated, considering if it is a suitable method for evaluating the anti-corrosion behavior of HDG rebar in concrete. By referring to the field performance of HDG rebar, which showed the corrosion of the substrate, and the previous study, which impressed the high rate of the acceleration condition, a comparatively low rate of the applied current density was used as a variable for experimentation. HDG rebar was embedded in w/c = 50% and 83.5% concrete, and the total current amount was impressed to corrode the full amount of zinc coating.

1. Concerning the observation of the surface change, a high ratio of w/c concrete made HDG rebar more easily corroded. Additionally, the further generation of zinc product did not proceed, after the corrosion of the substrate. The oxide film formed on HDG rebar showed a delaying effect of zinc corrosion in the initial stage, and it maintained a constant thickness, once it was fully formed.
2. As long as there was no corrosion of the substrate, HDG rebar showed a gradual increase of *R_p_*, as corrosion proceeded. Additionally, when the total current amount was the same, *R_p_* was relatively high in the lower applied current density. This behavior was mainly influenced by the apparent amount of zinc product, formed on the surface.
3. While the applied current density was different, if the w/c was the same, the actual corrosion amount was expected to be similar in this study. Additionally, even though there was some remaining zinc coating, once the corrosion of the substrate proceeded, HDG rebar stopped functioning the sacrificial anodic reaction of zinc.
4. The corrosion behavior of HDG rebar, which appeared in the acceleration test, was found to be similar to that in the actual corrosion environment. While the remaining zinc thickness at the time that the corrosion of the substrate started was thicker than the result of the field performance, a linear relationship was confirmed between the applied current density and the remaining zinc thickness. The higher the applied current density, the earlier the termination of the anti-corrosion effect, which caused a higher remaining zinc thickness. Therefore, in this study, it is recommended that the acceleration test be carried out below 0.1 mA/cm^2^.
5. In conclusion, while further experimental validation at various applied current densities is necessary, the acceleration test using impressed anodic current is expected to be appropriate for evaluating the durability of corroded HDG rebar, while the zinc coating is causing an anti-corrosion effect.

## Figures and Tables

**Table 1:** Specimen list and experimental variables.

No		Specimen	W/C (%)	Applied Current Density (mA/cm^2^)	Surface Area of HDG Rebar (cm^2^)	DCA^2^ (%)
1	1-1	W50-D160	50	0.16	62.5	25
	1-2					50
	1-3					75
	1-4					100
2	2-1	W50-D80		0.08	125	25
	2-2					50
	2-3					75
	2-4					100
3	3-1	W50-D20		0.02	500	25
	3-2					50
	3-3					75
4	4-1	W83.5-D80	83.5	0.08	125	15
	4-2					25
	4-3					50
	4-4					100
5	5-1	W83.5-D230-1 ^1^		0.23	43.5	10
	5-2					25
	5-3					50
	5-4					85
	5-5					100
6	6-1	W83.5-D230-2 ^1^				25
	6-2					50
	6-3					85

^1^ W83.5-D230-1 and W83.5-D230-2 are identical specimens; ^2^ DCA: Designed corrosion amount.

**Table 2:** The relationship among DCA, zinc coating thickness and the total current amount.

DCA (%)	Predicted Corrosion Thickness of Zinc Coating (μm)	Required Total Current Amount (mA × h/cm^2^)
10	18	10.53
15	27	15.79
25	45	26.32
50	90	52.63
75	135	78.95
85	153	89.47
100	180	105.26

**Table 3:** Mix proportion of concrete for the specimen.

W/C (%)	Unit Weight (kg/cm^3^)					Air (%)	Slump Height (cm)	Slump Flow (cm)	Compressive Strength ^4^ (N/mm^2^)
	W	C	S ^1^	G ^2^	Ad ^3^				
50.0	185	370	796	943	1.85	3.0	18.9	32	32.4
83.5	193	232	947	857	2.32	4.1	19.5	34	21.8

^1^ Fine aggregate; ^2^ Coarse aggregate; ^3^ Admixture; ^4^ Compressive strength at 28 days.

**Table 4:** Electrochemical properties and amount of corrosion of specimens, after the acceleration test.

No	Specimen	DCA ^1^ (%)	*E_corr_*^2^ (V)	*R_p_*^3^ (KΩ × cm^2^)	*i_corr_*^4^ (μA/cm^2^)	*t_remained_*^5^ (μm)	*t_corroded_*^6^ (μm)	Cracks on Concrete ^7^
1-1	W50-D160	25	−0.830	11.77	5.43	203.70	−54.29	Unfound
1-2		50	−0.963	13.50	4.74	188.37	−50.03	
1-3		75	−0.707	18.79	3.41	180.26	−36.09	
1-4		100	−0.590	27.53	2.32	159.11	−6.27	
2-1	W50-D80	25	−0.916	27.23	2.35	180.10	−40.33	
2-2		50	−0.810	41.88	1.53	178.12	−52.92	
2-3		75	−0.833	125.80	0.51	188.77	−55.62	
2-4		100	−0.957	77.29	0.83	154.84	−16.12	
3-1	W50-D20	25	−0.489	53.82	1.19	187.16	−48.30	
3-2		50	−0.397	69.47	0.92	170.08	−34.06	
3-3		75	−0.357	111.77	0.57	168.59	−31.86	
4-1	W83.5-D80	15	−0.882	19.63	2.87	123.60	23.31	Unfound
4-2		25	−0.931	23.26	2.42	111.43	37.40	
4-3		50	−0.644	56.81	0.99	89.95	70.05	Found
4-4		100	−0.549	69.13	0.81	97.63	79.07	Found
5-1	W83.5-D230-1	10	−0.463	10.20	5.52	Not measured		Unfound
5-2		25	−0.901	14.66	3.84	134.78	20.29	
5-3		50	−0.960	26.23	2.15	115.32	43.74	Found
5-4		75	−0.879	20.06	2.81	98.56	82.81	Found
5-5		100	−0.758	6.23	9.04	102.95	79.90	Found
6-1	W83.5-D230-2	20	−0.957	15.54	3.62	118.94	7.11	Unfound
6-2		50	−0.813	21.66	2.60	132.85	−0.73	
6-3		75	−0.884	158.00	0.36	101.00	52.44	Found

^1^ Designed corrosion amount; ^2^ Corrosion potential; ^3^ Polarization resistance; ^4^ Corrosion current density. In calculating *i_corr_*, the average tafel constant (*B*) was used, which is 0.063 V on w/c = 50% specimens and 0.056 V on w/c = 83.5% specimens; ^5^ Remaining coating thickness (= W_1_ − W_2_); ^6^ Estimated corrosion thickness of zinc coating (= W_0_–W_1_). (−) indicates the increase of thickness from the initial (uncorroded) state; ^7^ A crack, which appeared longitudinally with the rebar and broke the concrete surface.
